# WINFOCUS worldwide survey on central venous catheter insertion and position confirmation practices (CVC-ICON study)

**DOI:** 10.1186/s13089-025-00429-1

**Published:** 2025-08-14

**Authors:** Francesco Corradi, Giada Cucciolini, Guido Tavazzi, Adrian Wong, Cosmin Balan, Lawrence A. Melniker, Arif Hussain, Julina Md Noor, Jacob John Bailey, Anselmo A. Abdo Cuza, Alberto Goffi, Gabriele Via

**Affiliations:** 1 https://ror.org/03ad39j10 grid.5395.a 0000 0004 1757 3729 Department of Surgical, Medical, Molecular Pathology and Critical Care Medicine, Azienda Ospedaliero Universitaria Pisana, University of Pisa, Via Paradisa, 2, 56124 Pisa, PI Italy; 2 https://ror.org/00s6t1f81 grid.8982.b 0000 0004 1762 5736 Department of Clinical Surgical, Diagnostic and Pediatric Sciences, University of Pavia, Pavia, Italy; 3 https://ror.org/05w1q1c88 grid.419425.f 0000 0004 1760 3027 Anaesthesia, Intensive Care and Pain Therapy, Fondazione IRCCS Policlinico San Matteo, Pavia, Italy; 4 https://ror.org/044nptt90 grid.46699.34 0000 0004 0391 9020 Department of Critical Care, King’s College Hospital, London, UK; 5 1st Department of Cardiovascular Anesthesia and Intensive Care Medicine, Prof. Dr. C.C. Iliescu Institute for Emergency Cardiovascular Diseases, 022328 Bucharest, Romania; 6 https://ror.org/05bnh6r87 grid.5386.8 000000041936877X Department of Emergency Medicine NewYork-Presbyterian Brooklyn Methodist Hospital Weill College of Medicine, Cornell University, NewYork, USA; 7 https://ror.org/009p8zv69 grid.452607.2 0000 0004 0580 0891 King Abdulaziz Medical City, King Abdullah International Medical Research Center, Riyadh, Saudi Arabia; 8 https://ror.org/05n8tts92 grid.412259.9 0000 0001 2161 1343 Dept of Emergency Medicine, Universiti Teknologi MARA, Shah Alam, Selangor Malaysia; 9 https://ror.org/03dbr7087 grid.17063.33 0000 0001 2157 2938 Department of Medicine, Temerty Faculty of Medicine, University of Toronto, Toronto, Canada; 10 Medical-Surgical Research Center (CIMEQ), Havana, Cuba; 11 https://ror.org/03dbr7087 grid.17063.33 0000 0001 2157 2938 Interdepartmental Division of Critical Care Medicine, University of Toronto, Toronto, ON Canada; 12 https://ror.org/04skqfp25 grid.415502.7 . Michael’s Hospital, Unity Health Toronto, Toronto, ON Canada; 13 https://ror.org/04skqfp25 grid.415502.7 Keenan Research Centre, Li Ka Shing Knowledge Institute, Toronto, ON Canada; 14 https://ror.org/03c4atk17 grid.29078.34 0000 0001 2203 2861 Istituto Cardiocentro Ticino – Ente Ospedaliero Cantonale (EOC), Università Della Svizzera Italiana (USI), Lugano, Switzerland

**Keywords:** Patient safety, Hospital resources, Radiation exposure, Low-resource settings

## Abstract

**Background:**

Central venous catheters (CVC) are essential in medicine for monitoring, drug and fluid administration, and renal replacement therapy. Complications such as arrhythmias, endothelial damage, thrombosis, or hemothorax might arise from incorrect positioning. Despite evidence showing their reduction using ultrasound to guide insertion and correct tip positioning, and greater accuracy for tip position assessment vs. chest-X-ray (CXR), ultrasound adoption greatly varies worldwide. This study, conducted by the World Interactive Network Focused On Critical Ultrasound (WINFOCUS) aimed to assess global practices in CVC insertion and tip position confirmation.

**Methods:**

A web-based survey was conducted (April–September 2023) among WINFOCUS members/affiliates across five continents. It assessed clinical backgrounds, CVC insertion and tip position check methods, and reasons for not using ultrasound. Developed by WINFOCUS Research sub-committee, the survey was emailed, with two reminders. Data were analyzed using SPSS 27.0.

**Results:**

A total of 1,227 respondents (5.1% response rate) participated, mainly from Europe (33.5%), Asia (28.3%), and the Americas (30.9%), with 95.4% being physicians. Over half (51.3%) had over six years of experience and placed over 200 CVC, mostly using ultrasound guidance (70% of cases). The internal jugular vein (IJV) was the preferred insertion site (74%). Ultrasound was used for pre-insertion assessment (55%) and vessel puncture (57%) but less for guidewire confirmation (44%). CXR remained the primary method for tip position assessment (52%), while only 12% relied solely on bedside ultrasound. Barriers to exclusive ultrasound use included institutional guidelines (33.9%) and medico-legal concerns (13.8%).

**Conclusions:**

Despite evidence favoring ultrasound for CVC insertion and tip position confirmation, its use remains inconsistent, with CXR still widely used. This survey underscores the need for standardized protocols and training to enhance US adoption, improve patient safety, and reduce CXR reliance.

**Supplementary Information:**

The online version contains supplementary material available at 10.1186/s13089-025-00429-1.

## Background

Central venous catheters (CVC) are widely used in medical practice, with millions being inserted annually worldwide[1]. They are used mostly for hemodynamic monitoring, drug administration, fluid management, and renal replacement therapy.

Like with any other medical procedure, complications can occur. Some of them result from incorrect tip position, which may lead to severe complications within cardiac chambers (e.g., arrhythmias/cardiac wall damage)[2] or in the venous system (e.g., endothelial damage with extravasation, hemothorax, or thrombosis)[3, 4] or in the lung (pneumothorax). The use of ultrasound (US) during catheter insertion and tip position verification—ensuring placement within the distal 3 cm of the superior vena cava (SVC) before its junction with the right atrium (SVC-RA-J)[2]—significantly reduces these risks. Consequently, current guidelines recommend US for CVC placement[5, 6].

Trans-esophageal echocardiography (TEE) is currently the most accurate method to confirm the position of the CVC tip, as it can directly visualize the superior vena cava. However, its use is limited by its invasiveness, as well as the need for specific equipment and trained personnel. Therefore, a post-procedural chest-X-ray (CXR) is commonly performed after CVC cannulation of the upper extremities. But this imaging modality provides only indirect rather than direct visualization of the vessels, as it determines catheter tip position based on its projection onto anatomical landmarks, such as the carina or dorsal vertebrae. Conversely, Point-of-care ultrasound (PoCUS) is commonly used in everyday clinical practice by many physicians, and it has proven to be an effective alternative to TEE for CVC tip identification when contrast enhancement (CE) is used. Notably, it has demonstrated significantly higher diagnostic accuracy compared to CXR[7].

Despite recent evidence confirming the usefulness and reliability of US for CVC insertion and tip position verification, its use is not universal[6]. Significant geographical variations in US utilization have been reported[8, 9], and the practical aspects of this US application remain inconsistent at present. To address the gap between scientific evidence and clinical practice, the World Interactive Network Focused On Critical Ultrasound (WINFOCUS) initiated an international audit that aims to delineate global practices regarding CVC insertion and confirmation of correct placement, with a particular emphasis on the utilization of US. The findings of this survey will also guide the design and implementation of a multinational prospective observational study investigating the use and outcomes associated with CVC placement.

## Materials and methods

### Methods

A web-based cross-sectional survey was distributed between April 4 and September 9, 2023, through WINFOCUS’s network in five continents. This study was deemed exempt from review by the local ethics committee of Pisa. The WINFOCUS Research sub-committee was responsible for developing the survey. The questionnaire included four subdomains: (1) general information, (2) volume and characteristics of the procedures, (3) methods for performing CVC cannulation and verifying CVC tip position, and (4) reasons for non-use of US as a position checking method. The survey was constructed using the SurveyMonkey online platform (SurveyMonkey Inc., San Mateo, California, USA) and piloted and tested as per current recommendations[10]. The survey was sent individually by e-mail to all WINFOCUS members/affiliates. Two reminders were sent. The information was collected anonymously and stored on a secure digital data collection platform.

### Statistical analysis

The reported approaches to CVC placement and tip position verification were analyzed across different medical specialties. Descriptive statistics were used to summarize the data. Results were expressed as median ± IQR for continuous variables. A *p-*value < 0.05 was considered to indicate statistically significant differences. Data were analyzed using SPSS 27.0. A colored world map graph was built using the ggplot2 and Naturalearth software packages in R[11–13].

## Results

### Study population and ultrasound use frequency

One thousand two hundred and forty-six forms were submitted to the online data platform (5.1% response rate), and 1.227 (98.5%) individuals confirmed their agreement to participate. The characteristics of the responding health care professionals (95.4% physicians) are presented in Table 1**.**

**Table 1:** Demographics of the 1.227 Respondents to the survey

What is your current profession?	Numbers	Percentages
Physician	997	95.4
Physician Assistant	23	2.2
Nurse	11	1.1
Paramedic/Emergency Medical Technician	8	0.8
Nurse practitioner	4	0.4
Sonographer	2	2

What is your specialty?	Numbers	
Critical care Medicine	501	27
Anaesthesiology	407	21.9
Non-Cardiovascular Anaesthesiology	334	18
Emergency Medicine	265	14.3
General Surgery	19	8.6
Internal Medicine—Hospitalist	93	5
Cardiovascular Anaesthesiology	73	3.9
Pediatrics	43	3
Family Medicine	15	2.4
Nephrology	28	2
Cardiology	22	1.4
Internal Medicine Primary Care	20	1.1
Respirology/Pulmonary Medicine	12	0.6
Vascular Surgery	9	0.4
Radiology/Medical Imaging	7	0.4
Cardiac Surgery	5	0.3
Thoracic Surgery	3	0.1

Does your institution have a dedicated vascular access team?	Numbers	Percentages
Yes	362	34.8
No	679	65.2

How many years have you been in independent practice?	Numbers	Percentages
Still in training	145	11.6
≤3	111	8.9
3–5	141	11.3
6–10	220	17.7
11–20	258	20.7
> 20	167	13.4

How many central lines have you personally placed (independently or under supervision) during your medical career?		
	Numbers	Percentages
≤50	164	16.4
51–100	159	15.9
101–200	164	16.4
> 200	513	51.3

When considering all the CVC you have personally inserted during your career what percentage of them was US guided or assisted?		
	Numbers	Percentages
Median (IQR and ranges)	70%	IQR: 40–90; Ranges: 0–100
0%	25	2.5
< 10%	42	4.2
10–20%	67	6.7
21–30%	77	7.3
31–40%	43	4.3
41–50%	118	11.8
51–60%	56	5.6
61–70%	74	7.4
71–80%	133	13.5
81–90%	138	13.8
91–99%	106	10.6
100%	121	12.1

How many central lines have you personally placed (independently or under supervision) in the last year?		
< 10	191	19.2
11–20	250	25.2
21–50	298	30
> 100	102	10.3

When considering all the CVC you have personally inserted in the last year what percentage of them was US guided or assisted?		
Median (IQR and ranges)	97%	IQR: 68–100; Ranges: 0–100
0%	40	4
< 10%	73	7.3
10–20%	45	4.5
21–30%	37	3.7
31–40%	28	2.8
41–50%	55	5.5
51–60%	28	2.8
61–70%	27	2.7
71–80%	74	7.4
81–90%	91	9.1
91–99%	108	16.5
100%	458	46.1

**Fig. 1:**
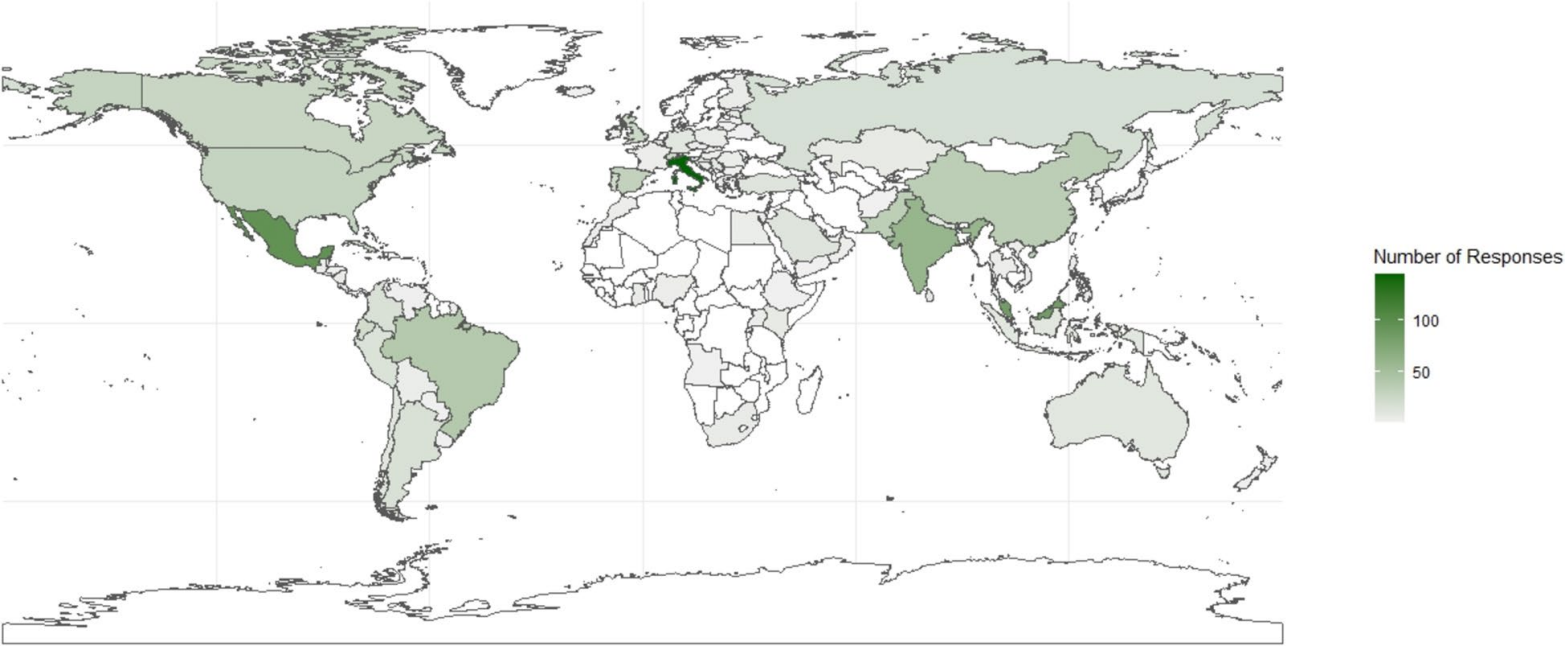
Number of respondents by country

**Fig. 2:**
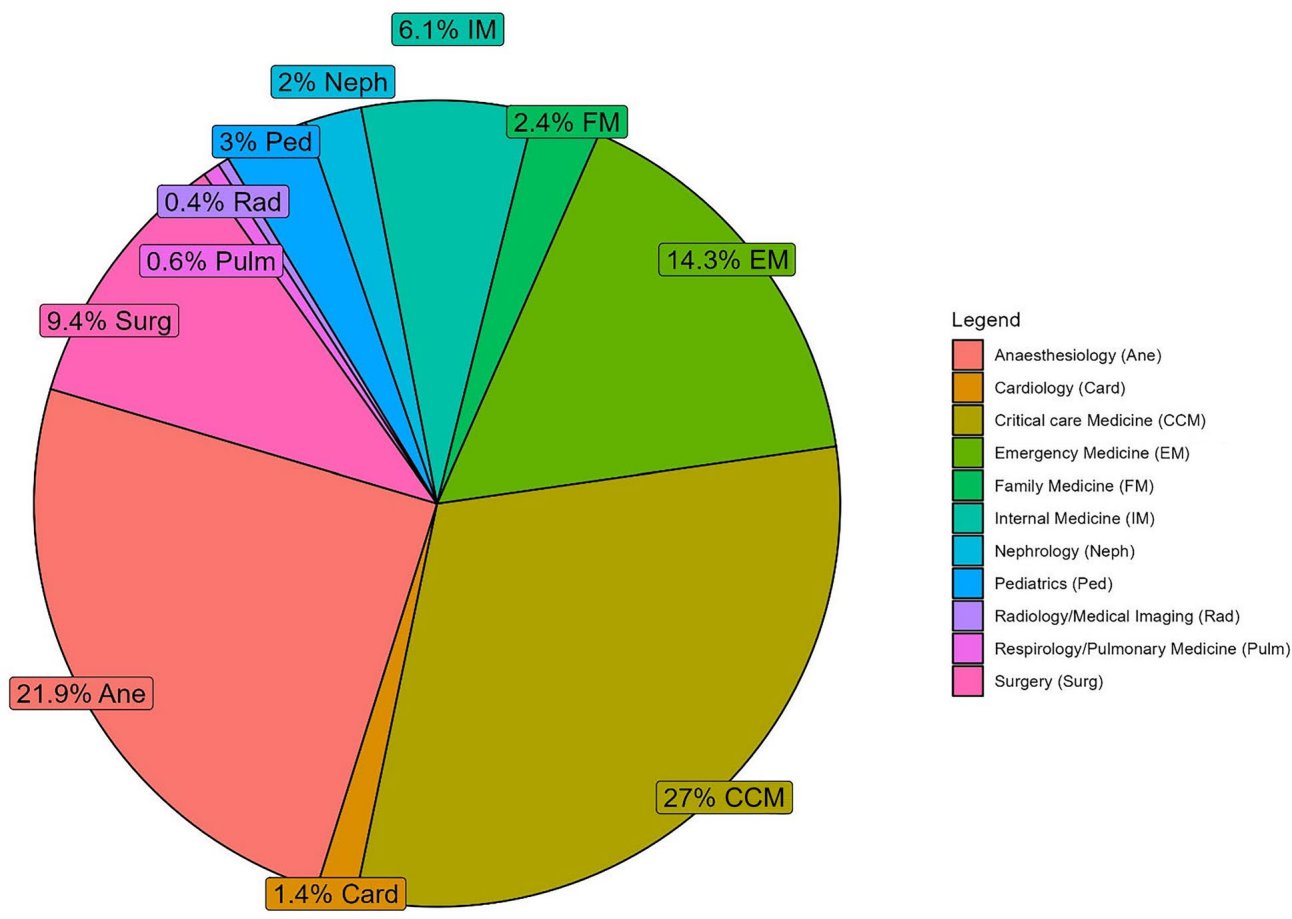
Percentage of healthcare professionals responding to the survey, according to specialty

Respondents were from the following continents: Europe (33.5%), Asia (28.3%), the Americas (30.9%), Africa (5.8%), and Oceania (1.5%). (Fig. 1) A total of 1,131 (92.2%) respondents reported inserting CVC as part of their clinical practice. The most represented specialties were critical care medicine (n:501; 27%), anesthesiology (n:407; 21.9%), and emergency medicine (n:265; 14.3%). (Fig. 2) The majority of respondents had more than six years of independent clinical practice (n:645; 51.8%) and had personally placed over 200 CVC during their medical careers (n:513; 51.3%), either independently or under supervision.

Participants reported a median US utilization rate of 70% (IQR: 40–90) for central venous catheter (CVC) insertions throughout their careers. While 12.1% consistently used US for all insertions, 6.7% relied on it for fewer than 10% of procedures, and 2.5% never used it. In the last year, a significant increase in US use was observed, with a median utilization rate of 97% (IQR: 68–100). In this same period, nearly half of respondents (46.1%) employed US for every insertion, whereas only 7.3% used it in fewer than 10% of cases.

### Preferred CVC insertion site

The preferred site for CVC insertion was the internal jugular vein (IJV) (74%), followed by the subclavian vein (SV) (17%). (Table 2) Only a minority of respondents preferred the femoral vein (FV) (6%) or peripherally inserted central catheters (PICC) (3%).

**Table 2:** Preferred approach for CVC positioning

Which is the CVC approach you favor/perform the most?	Numbers	Percentages
Internal jugular vein	675	74.4
Subclavian vein	153	16.9
Femoral vein	50	5.5

Which technique do you preferentially use when inserting internal jugular vein CVC with US?		
Out-of-plane approach	562	63.2
In-plane approach	327	36.8

Which technique do you preferentially use when inserting subclavian vein CVC with US?		
Out-of-plane approach	236	35.8
In-plane approach	423	64.2

Which technique do you preferentially use when inserting femoral vein CVC with US?		
Out-of-plane approach	552	69.7
In-plane approach	240	30.3

Which technique do you preferentially use when inserting PICCs with US?		
Out-of-plane approach	204	61.8
In-plane approach	126	32.2

*Abbreviations*: CVC: central venous catheter; US: ultrasounds

### Insertion technique

The use of US was part of the standard of care for pre-insertion anatomical assessment in 54.6% of cases, for needle advancement guidance during vessel puncture in 57.2% of cases, and only in 43.8% of cases after guidewire insertion to confirm correct placement in the vein.

The preferred US technique for CVC insertion was reported to be the out-of-plane approach for IJV (63%), the FV (70%), or PICC catheters (62%). Conversely, most respondents preferred the in-plane approach for the SV cannulation (64%). (Table 3).

**Table 3:** Insertion Technique

When inserting CVC, how often do you use ultrasound for pre-insertion anatomy assessment?	Numbers	Percentages
Never	30	3.1
Only as rescue when landmark approach has failed	32	3.3
Rarely (< 10%)	35	3.7
Sometimes (10–50%)	69	7.2
Most of the time (51–75%)	91	9.5
Very Often (76–99%)	177	18.5
Always (100%)	522	54.6

When inserting CVC, how often do you use ultrasound during advancement of the needle for vessel puncture?		
Never	40	4.2
Only as rescue when landmark approach has failed	35	3.7
Rarely (< 10%)	32	3.4
Sometimes (10–50%)	70	7.3
Most of the time (51–75%)	80	8.4
Very Often (76–99%)	151	15.8
Always (100%)	545	57.2

When inserting CVC, how often do you use ultrasound after guidewire insertion to confirm placement in the vein and only in the vein?		
Never	66	6.9
Only as rescue when landmark approach has failed	32	3.4
Rarely (< 10%)	91	9.5
Sometimes (10–50%)	96	10.1
Most of the time (51–75%)	97	10.2
Very Often (76–99%)	154	16.2
Always (100%)	417	43.8

*Abbreviations*: CVC: central venous catheter

### Confirmation of the appropriate CVC placement in the venous system of the upper extremities

To confirm that the CVC is correctly positioned in a vein (Table 4), the majority of respondents (52%) reported “always” using CXR typically in combination with other methods. The next most common technique was US visualization of the guidewire within the vein (45%), followed by saline or fluid injection (23%), observation of low-pressure blood return in the line (22%), intravascular electrocardiography (8%), blood gas analysis (6%), US contrast injection (4%), central venous pressure transduction (4%), post-insertion tip visualization via TEE (3%), and fluoroscopy (0.6%). Notably, 2.5% of respondents stated they do not routinely verify CVC positioning in the upper extremities, considering it unnecessary. Only 12% relied solely on bedside US to confirm proper CVC placement. (Table 4).

**Table 4:** Confirmation of appropriate placement of CVC in the venous system of the upper extremities

Which method do you use and how often do you use it to confirm appropriate placement of CVC in the venous system (i.e., not in an artery or subcutaneous tissue) of the upper extremities?		
Chest-X-Ray	Numbers	Percentages
Never	44	5.4
Only as rescue when landmark approach has failed	23	2.8
Rarely (< 10%)	48	5.9
Sometimes (10–50%)	63	7.8
Most of the time (51–75%)	69	8.5
Very Often (76–99%)	143	17.7
Always (100%)	419	51.8

Visualization of wire in venous vessel with ultrasound	Numbers	Percentages
Never	62	7.8
Only as rescue when landmark approach has failed	15	1.9
Rarely (< 10%)	67	8.4
Sometimes (10–50%)	80	10
Most of the time (51–75%)	69	8.6
Very Often (76–99%)	143	17.9
Always	363	45.4

Intravascular electrocardiogram (ECG)	Numbers	Percentages
Never	504	64.1
Only as rescue when landmark approach has failed	13	1.7
Rarely (< 10%)	96	12.2
Sometimes (10–50%)	57	7.3
Most of the time (51–75%)	28	3.6
Very Often (76–99%)	27	3.4
Always	61	7.8

Injection of saline/fluid	Numbers	Percentages
Never	249	61.7
Only as rescue when landmark approach has failed	22	2.8
Rarely (< 10%)	89	11.3
Sometimes (10–50%)	104	13.2
Most of the time (51–75%)	56	7.1
Very Often (76–99%)	86	10.9
Always	180	22.9

Injection ultrasound contrast (e.g., air-saline mixture, commercially available contrast-enhancing agent)		
	Numbers	Percentages
Never	585	74.2
Only as rescue when landmark approach has failed	27	3.4
Rarely (< 10%)	57	7.2
Sometimes (10–50%)	49	6.2
Most of the time (51–75%)	12	1.5
Very Often (76–99%)	23	2.9
Always	35	4.4

Blood gas analysis	Numbers	Percentages
Never	263	42
Only as rescue when landmark approach has failed	100	12.7
Rarely (< 10%)	189	23.9
Sometimes (10–50%)	124	15.7
Most of the time (51–75%)	30	3.8
Very Often (76–99%)	40	5.1
Always	44	5.6

Central venous pressure transduction	Numbers	Percentages
Never	394	50.1
Only as rescue when landmark approach has failed	69	8.8
Rarely (< 10%)	134	17
Sometimes (10–50%)	91	11.6
Most of the time (51–75%)	26	3.3
Very Often (76–99%)	38	4.8
Always	35	4.4

Demonstration of blood tracking back at low pressure in the line (venous flow)	Numbers	Percentages
Never	222	27.8
Only as rescue when landmark approach has failed	45	5.6
Rarely (< 10%)	99	12.4
Sometimes (10–50%)	95	11.9
Most of the time (51–75%)	77	9.6
Very Often (76–99%)	82	10.3
Always	178	22.3

CVC tip visualization post-insertion using TEE	Numbers	Percentages
Never	604	76.6
Only as rescue when landmark approach has failed	31	3.9
Rarely (< 10%)	67	8.5
Sometimes (10–50%)	37	4.7
Most of the time (51–75%)	17	2.2
Very Often (76–99%)	12	1.5
Always	20	2.5
Very Often (76–99%)	118	14.4
Always	100	12.2

Fluoroscopy	Numbers	Percentages
Never	652	82.1
Only as rescue when landmark approach has failed	29	3.7
Rarely (< 10%)	64	8.1
Sometimes (10–50%)	23	2.9
Most of the time (51–75%)	8	1
Very Often (76–99%)	13	1.6
Always	5	0.6

No confirmatory step required	Numbers	Percentages
Never	607	80.9
Only as rescue when landmark approach has failed	16	2.1
Rarely (< 10%)	45	6
Sometimes (10–50%)	42	5.6
Most of the time (51–75%)	8	1.1
Very Often (76–99%)	13	1.7
Always	19	2.5

Excluding femoral vein CVC, how often do you use bedside US as the only method to confirm appropriate CVC placement?		
	Numbers	Percentages
Never	169	20.7
Only when CXR is not readily available or in case of urgent need to start infusion	129	15.8
Rarely (< 10%)	101	12.3
Sometimes (10–50%)	117	14.3
Most of the time (51–75%)	84	10.3
Very Often (76–99%)	118	14.4

*Abbreviations*: CVC: central venous catheter

### Techniques to identify the position of the CVC tip at the cavo-atrial junction

Among respondents, 5.7% indicated that they do not routinely verify the position of the CVC tip. Of the remaining 94.3%, the most commonly used method was CXR (44.9%), followed by echocardiography (24.5%), either transthoracic or transesophageal. Additional techniques included measuring the distance from the puncture site to an anatomical landmark at the cavo-atrial junction (11.4%), intravascular electrocardiography (5.6%), use of a calculated formula to estimate the required catheter length for proper tip placement (6%), and fluoroscopy (1.8%).

Among those utilizing US to visualize the catheter tip at the SVC–right atrium (RA) junction, various approaches were reported: contrast-enhanced US (using bubble detection in the RA via PoCUS or TEE) (8.2%), direct visualization of the catheter tip in the SVC-RA junction using PoCUS apical view (7.3%), or PoCUS bicaval subcostal view with confirmation via contrast medium at the exit point (6.4%). Additionally, 2.6% used TEE for direct catheter tip visualization. (Table 5).

**Table 5:** Techniques to identify the position of the CVC TIP at the CAVO-atrial junction

Which technique do you use to identify the position of the TIP of CVC at the cavo-atrial junction?	Numbers	Percentages
CXR	640	44.9
I measure the distance between identified puncture site and the anatomical landmark for the cavo-atrial junction	162	11.4
Ultrasound contrast medium injection, looking for bubbles reaching the right atrium within a certain time from the injection either with TTE or TTE	117	8.2
Visualization of the catheter tip in SVC-RA using TTE	105	7.3.
Visualization of the catheter tip in SVC-RA using TTE subcostal view, and confirmed by visualization of the contrast medium exit point	91	6.4
I use a formula to predict the required length to position the catheter tip at the cavo-atrial junction	86	6
I do not routinely check the position	82	5.7
Intravascular electrocardiogram	80	5.6
Visualization of the catheter tip in SVC-RA using TEE	38	2.6
Fluoroscopy	25	1.8

*Abbreviations*: CXR: chest-X-ray; CVC: central venous catheter; SVC-RA: superior vena cava-to-right atrium junction; US: ultrasound; TEE: transesophageal echocardiography; TTE: transthoracic echocardiography

**Fig. 3:**
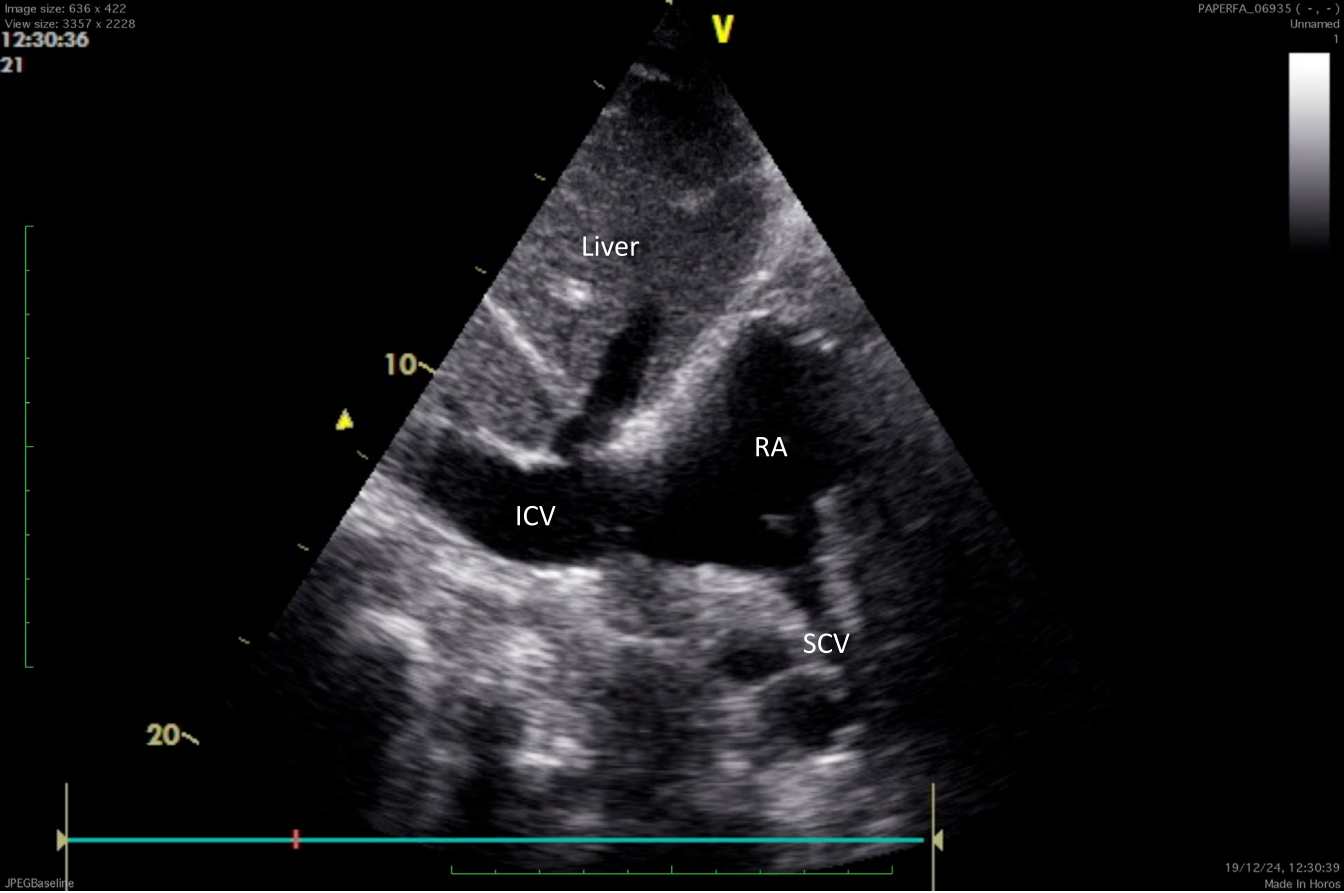
Representation of the Superior Vena Cava-to Right Atrium visualization through the trans-thoracic bicaval subcostal view

Among practitioners using the PoCUS bicaval subcostal view (Fig. 3), 9% did not use any contrast medium, 34% used saline only, and 57% used a combination of air, blood, and saline or dedicated US contrast agents. Overall, fewer than half of respondents (44.5%) used some form of contrast medium to confirm catheter tip positioning. (Table 6).

**Table 6:** Ultrasound contrast medium to CONFIRM CATHETER TIP LOCATION

What type of ultrasound contrast medium do you use to confirm catheter tip location?		
	Numbers	Percentages
I never use ultrasound contrast	499	55.5
Air-saline mixture	178	178
Saline	155	17.2
Air-saline-blood mixture	45	5
Air-blood mixture	12	1.3
Commercially available contrast-enhancing agent (e.g. SonoVue; Lumason; Definity; Optison; Imavist)	10	1.1

### Factors preventing the use of US as the sole method to confirm CVC placement

The main barrier to using US as the sole method for confirming CVC placement, reported by 33.9% of respondents, is the requirement to follow local institutional/departmental protocols that still mandate CXR. The second most cited concern was medico-legal liability (13.8%), followed by insufficient US proficiency (8.8%). (Table 7).

**Table 7:** Factors preventing the use of US as the SOLE method to CONFIRM CVC PLACEMENT

Are there any factors that prevent you from using US as the only method to confirm placement of a CVC		
	Numbers	Percentages
My institution has a policy or protocol requiring CXR after central line placement	298	298
No barriers, I currently routinely use ultrasound and NOT chest radiography for CVC confirmation	179	20.3
Medico-legal concerns	121	13.8
I lack sufficient US confidence to use this technique	77	8.8
I wasn't aware/ didn't appreciate that this was an option	58	6.6
Limited access to US system	56	6.3
US is not as sensitive as CXR to confirm proper CVC position	46	5.2
It is more convenient/easier to obtain a CXR	45	5.1

*Abbreviations*: CXR: chest-X-ray; CVC: central venous catheter; US: ultrasounds

### Routine methods used to exclude procedure-related pneumothorax

To rule out procedure-related pneumothorax, 53% of respondents reported using both CXR and US, while 31% relied solely on CXR and 15% used only US. The two most frequently cited reasons for not using US exclusively to detect or exclude pneumothorax after CVC placement were institutional protocols mandating CXR and medico-legal concerns. (Table 8).

**Table 8:** Routine methods used to exclude a procedure-related pneumothorax and factors preventing the use of US as the only method to identify/exclude pneumothorax after placement of a CVC

What is your routine method to exclude a procedure-related PNX after a central line has been placed?	Numbers	Percentages
US and CXR	421	52.7
CXR alone	246	30.8
US alone	119	14.9
Auscultation alone	10	1.3
Auscultation and CXR	1	0.1
Auscultation and US	1	0.1
Auscultation and US and CXR	1	0.1

Are there any factors that prevent you from using US as the ONLY method to identify/exclude PNX after placement of a CVC?		
My institution has a policy or protocol requiring CXR after central line placement	385	33.1
No barriers, I currently routinely use ultrasound and NOT chest radiography for PTX detection post CVC insertion	172	14.8
As I use CXR for confirmation of CVC placement, I find CXR easily address both questions	170	14.6
Medico-legal concerns	166	14.3
I lack sufficient US confidence to use this technique	87	7.5
It is more convenient/easier to obtain a CXR	71	6.1
Limited access to US system	50	4.3
Ultrasound is not as sensitive as CXR in evaluation of PNX	41	3.5
I wasn't aware/ didn't appreciate that this was an option	21	1.8

*Abbreviations*: CXR: chest-X-ray; CVC: central venous catheter; PNX: pneumothorax; US: ultrasounds

## Discussion

This study represents the largest cross-sectional analysis of US use for CVC insertion and tip confirmation across various medical specialties, regions, and levels of expertise. The key finding is that, despite the majority of respondents (51.3%) being seasoned PoCUS practitioners with over six years of experience, adherence to recommended practices for CVC placement and tip verification remained low, irrespective of geographic location or medical specialty.

A standardized, protocol-driven approach for successful US-guided CVC insertion has been previously recommended[14], comprising four key steps: (1) confirming needle placement in the vein, (2) confirming guidewire position in the vein, (3) verifying correct catheter tip placement, and (4) ruling out procedure-related complications. However, our survey showed that only a small proportion of respondents consistently follow all four steps. Adherence was notably higher for the first two (57.2% and 45.4%, respectively), while compliance significantly declined for the third and fourth steps (6.4% and 14.9%, respectively). This may stem from the misconception that identifying and/or cannulating the vein ensures procedural success and minimizes the risk of complications. However, this approach should be discouraged, as it can result in delayed recognition of treatable, potentially life-threatening complications and increase the risk of adverse outcomes.

Our survey revealed a wide variety of techniques used to confirm proper CVC tip placement. Among imaging methods, CXR remained the most commonly used routine approach (44.9%), despite evidence showing its inferior performance compared to US. This is notable given that most respondents, based on their reported experience, can be considered skilled in US. A recent study found that CXR had a sensitivity of 32%, specificity of 93%, overall diagnostic accuracy of 73%, and weak agreement with the reference standard, TEE (k = 0.29). In contrast, contrast-enhanced transthoracic echocardiography (CE-TTE) using a subcostal view showed significantly better performance, with a sensitivity of 97%, specificity of 90%, diagnostic accuracy of 92%, and strong agreement with TEE (k = 0.79). Interestingly, CE-TTE using the apical four-chamber view was less effective in detecting CVC tip misplacements, with a sensitivity of 22%, specificity of 94%, diagnostic accuracy of 70%, and poor concordance with CE-TEE (k = 0.17), performing similarly to CXR in this context. The use of an agitated saline mixture is essential, as it facilitates a more precise determination of the CVC tip’s position at the SVC-RA junction than does 2D imaging alone. This result is expected, since CXR cannot directly visualize the SVC-RA junction and instead depends on projection of the catheter tip onto other anatomical landmarks, which can be unreliable[15, 16]. Similarly, the apical four-chamber view does not allow direct visualization of the SVC-RA junction. Conversely, CE-TTE using the subcostal view is the only non-invasive method capable of directly visualizing the position of the CVC tip in relation to vascular and cardiac structures [Additional files 1, 2, 3]. The injection of US contrast or agitated saline (“bubble test”) to evaluate flow patterns in the right atrium and/or to measure the interval between injection and bubble appearance has been suggested as a method to confirm proper CVC placement. However, we believe this technique merely confirms that the CVC is within the venous system, without providing accurate localization of the catheter tip[17]: the elapsed time can be influenced by various factors, including venous return, length and diameter of the CVC, and the lack of precise synchronization and speed of the injection. A previous study[18] reported that complete opacification of the right atrium following contrast injection occurred in only half of patients with misplaced CVC. Another study questioned the reliability of using predefined cut-off transition times to confirm the central catheter tip’s position[19]. Additionally, the type of contrast used is critical for accurately identifying the CVC tip location. Similarly, air–saline or air–blood–saline mixtures can be employed to detect foramen ovale patency [20, 21]. Among these mixtures, a composition of 80% saline, 10% air, and 10% blood has demonstrated superior efficacy in specific clinical scenarios[22, 23]. Air is highly echogenic due to its substantial acoustic impedance difference compared to blood, while adding blood to saline produces smaller, more uniform, and stable microbubbles. Despite these advantages, our survey revealed that fewer than 5% of respondents reported using the air–saline–blood mixture.

Finally, it is important to highlight the under-utilization of the subclavian site (16.9%), irrespective of medical specialty or clinical seniority, despite recommendations supporting its use to reduce the risk of infectious complications [24]. The limited use of subclavian access may partly be explained by a higher risk of pneumothorax and insufficient training during the pre-US era; however, the adoption of US guidance has significantly decreased the risk of mechanical complications[25]. Additionally, while most respondents preferred the “in-plane approach” for subclavian vein cannulation, the “out-of-plane” technique offers notable benefits regarding insertion time, success rates, fewer needle redirections, reduced skin punctures, and lower complication rates. [25]

## Limitations

Interpreting results from a multinational survey inherently involves methodological limitations, primarily due to the lack of patient-specific data. Our objective was to obtain a broadly representative sample of clinicians to provide insight into practice variations across international boundaries and among diverse professional groups. However, some countries may have been disproportionately represented. Additionally, since the survey was distributed via the WINFOCUS mailing list, respondents were likely biased toward US-guided practices. As a consequence, our findings may not fully reflect global clinical practices, and the actual use of CXR for confirming central vascular access may be greater than indicated by this survey. Rather than diminishing our findings, this consideration further strengthens our conclusions.

## Conclusions

The primary finding of this survey is the underutilization of US during the four steps of CVC placement. Although current evidence supports US use for both CVC insertion and tip position verification, international guidelines have yet to provide definitive recommendations. Consequently, many clinicians either do not utilize US or employ it only partially, continuing to rely on CXR as the standard for confirming catheter tip position[26]. We advocate for US to become the primary method for verifying catheter placement. To enhance patient safety, optimize resource utilization, and minimize radiation exposure, strategies aimed at reducing the routine use of CXR for confirming CVC tip position should be implemented. Furthermore, an ongoing priority remains the establishment of robust methodologies that support guideline recommendations endorsing US as the standard approach for CVC placement, reserving CXR for scenarios where US is not feasible.

## Supplementary Information

**Additional File 1**: Transthoracic subcostal acoustic window focussed on the superior vena cava-right atrium junction exit-point showing a clear jet flow coming from the right atrium immediately after agitated saline injection, corresponding to aberrant central line tip positioning.

**Additional File 2**: Transthoracic subcostal acoustic window focussed on the superior vena cava-right atrium junction exit-point showing a clear jet flow coming from the superior vena cava-to-right atrium junction immediately after agitated saline injection with the concomitant visualization of the catheter tip, corresponding to correct central line tip positioning.

**Additional File 3**: Transthoracic subcostal bicaval acoustic window showing superior vena cava-right atrium junction: a laminar flow appears from the superior vena cava after agitated saline injection, without the direct visualization of the catheter tip exit point. This condition only confirms the presence of the CVC in the venous system without providing precise tip localization and cannot rule-out high-lying CVC tip location eventually leading to severe complications due to secondary endothelial damage (extravasation, pleural effusion or thrombosis with infections).

## Data Availability

Data will be made available by the authors for global collaboration on reasonable request, within the national restrictions imposed by privacy laws and ethics.

## Abbreviations

CE: Contrast-enhancement
CVC: Central venous catheters
CXR: Chest-X-ray
FV: Femoral vein
ICU: Intensive care unit
IJV: Internal jugular vein
PICC: Peripherally inserted central catheters
SV: Subclavian vein
SVC: Superior vena cava
SVC-RA: Superior vena cava-right atrium junction
TEE: Transesophageal echocardiography
CE-TTE: Contrast-enhanced transthoracic echocardiography transthoracic echocardiography
US: Ultrasound
WINFOCUS: World Interactive Network Focused on Critical UltraSound
CVC-ICON: Central venous catheter insertion and position confirmation
PoCUS: Point-of-care UltraSound
