# Supplementary figures and images for: WINFOCUS worldwide survey on central venous catheter insertion and position confirmation practices (CVC-ICON study)
Source: Ultrasound J. 2025 Aug 14;17:41. doi: 10.1186/s13089-025-00429-1 (PMC12354427; doi:10.1186/s13089-025-00429-1)

## Slide 1
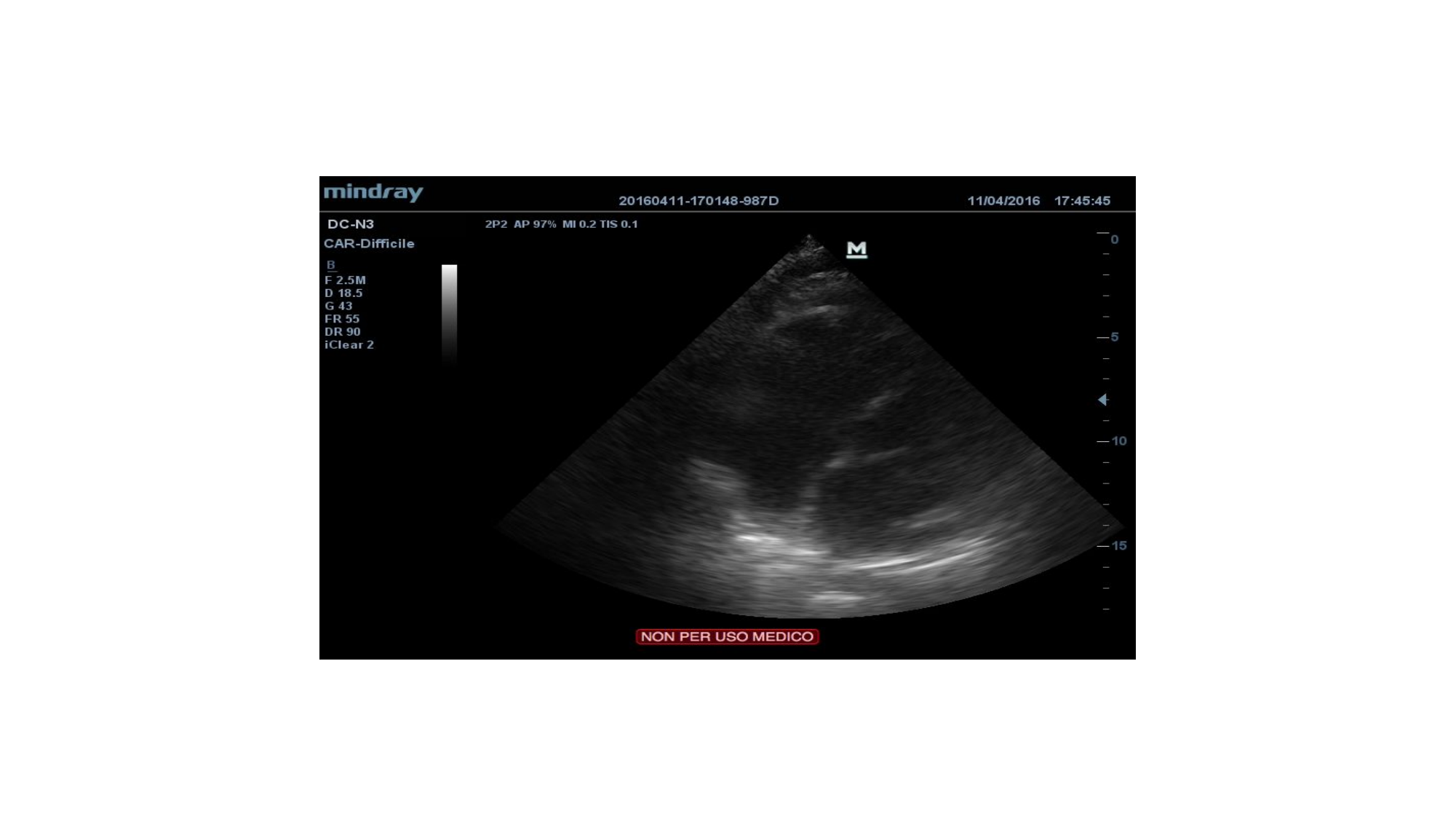

Supplement: Supplementary file 1 — Additional File 1: Transthoracic subcostal acoustic window focussed on the superior vena cava-right atrium junction exit-point showing a clear jet flow coming from the right atrium immediately after agitated saline injection, corresponding to aberrant central line tip positioning. [file 13089_2025_429_MOESM1_ESM.pptx]

## Slide 1
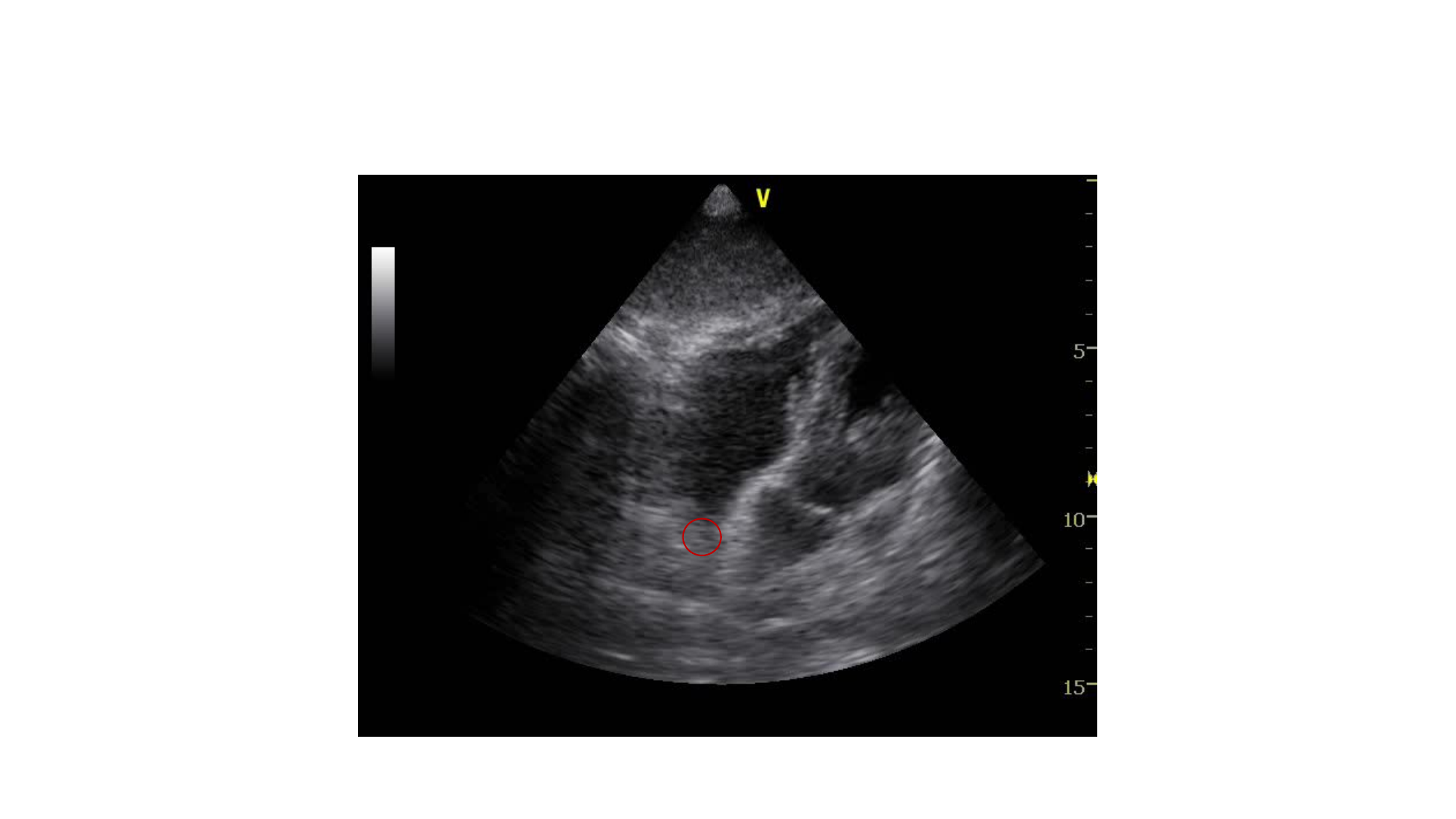

Supplement: Supplementary file 2 — Additional File 2: Transthoracic subcostal acoustic window focussed on the superior vena cava-right atrium junction exit-point showing a clear jet flow coming from the superior vena cava-to-right atrium junction immediately after agitated saline injection with the concomitant visualization of the catheter tip, corresponding to correct central line tip positioning. [file 13089_2025_429_MOESM2_ESM.pptx]

## Slide 1
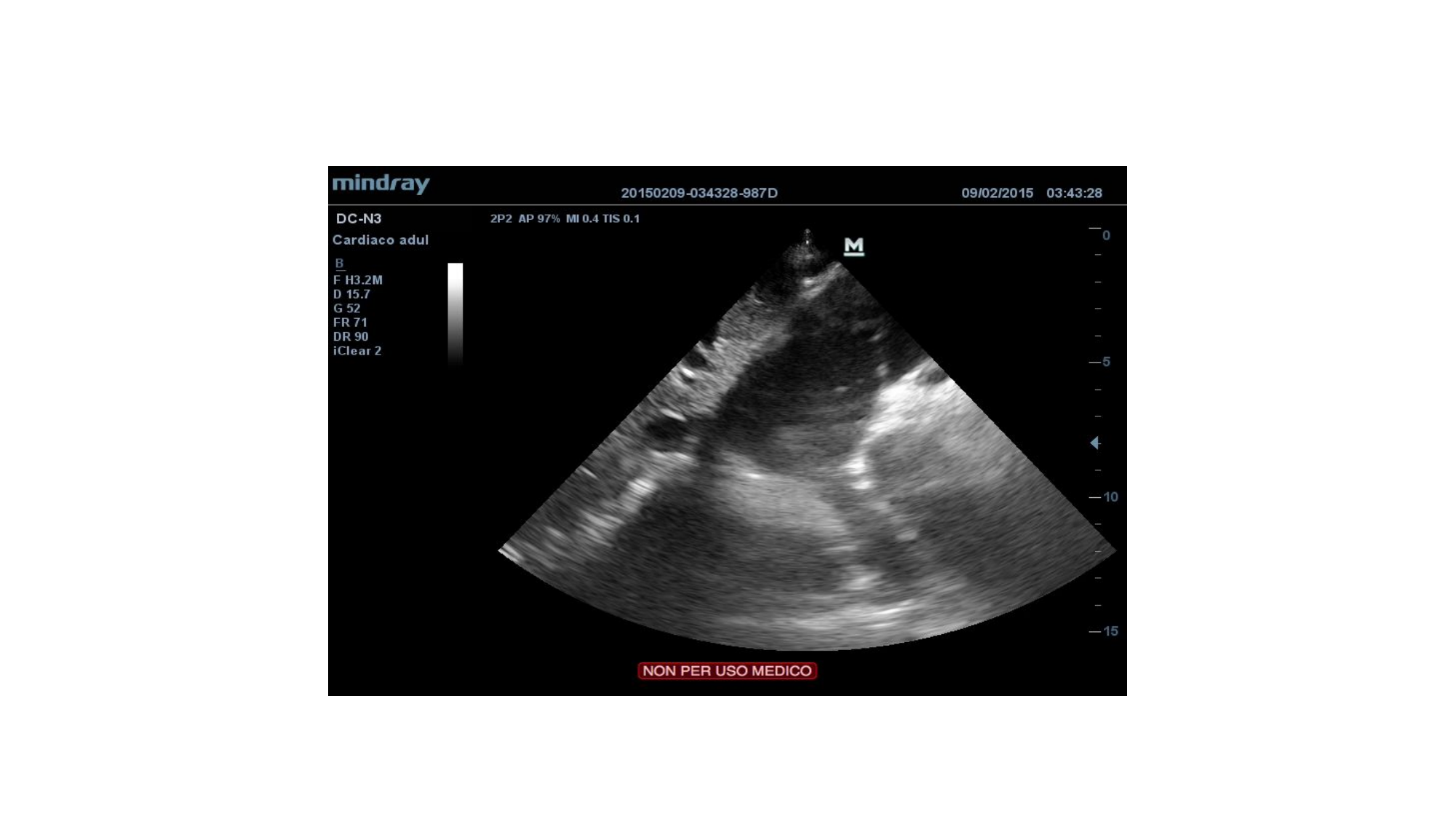

Supplement: Supplementary file 3 — Additional File 3: Transthoracic subcostal bicaval acoustic window showing superior vena cava-right atrium junction: a laminar flow appears from the superior vena cava after agitated saline injection, without the direct visualization of the catheter tip exit point. This condition only confirms the presence of the CVC in the venous system without providing precise tip localization and cannot rule-out high-lying CVC tip location eventually leading to severe complications due to secondary endothelial damage (extravasation, pleural effusion or thrombosis with infections). [file 13089_2025_429_MOESM3_ESM.pptx]
